# Food sources of energy and nutrients among Canadian adults following a gluten-free diet

**DOI:** 10.7717/peerj.9590

**Published:** 2020-07-27

**Authors:** Jennifer A. Jamieson, Anna Neufeld

**Affiliations:** Department of Human Nutrition, St. Francis Xavier University, Antigonish, NS, Canada

**Keywords:** Cereal, Grain, Gluten, Free, Diet, Celiac, Nutrient, Wheat, Pattern

## Abstract

**Background:**

The gluten-free diet (GFD) involves the elimination of wheat and related grains. Wheat is a key fortification vehicle for nutrients such as iron and B vitamins. While there is growing evidence of low nutrients intake and poor diet quality amongst people following long-term GFD, few studies have used a dietary pattern approach to analyse top food sources of nutrients in today’s complex food environment. Thus, the purpose of this study was to identify food sources of energy and nutrients from previously collected diet records of adults following a GFD.

**Methods:**

Three, 3-day food records were collected from 35 participants in a lifestyle intervention study (*n* = 240 records). All food items were categorised according to the Bureau of Nutritional Sciences Food Group Codes. Percentages of total dietary intakes from food groups were ranked.

**Results:**

Mean intakes of dietary fibre, calcium and iron (females) were lower than recommended, with half the sample consuming below the recommended proportion of energy as carbohydrate. Meat, poultry and fish were the top source of energy (19.5%) in the diet. Gluten-free (GF) grain products were the top source of carbohydrate, fibre and iron and second greatest source of energy. Amongst grains, breakfast/hot cereals, yeast breads, and mixed grain dishes were the greatest nutrient contributors, despite most commercial cereals and breads (65%) being unenriched. Legumes were not frequently consumed.

**Conclusions:**

GF grains were the top food source of carbohydrate, fibre and iron, despite few brands being enriched or fortified. It is a challenge to assess and monitor nutrient intakes on GFD due to the lack of nutrient composition data for B vitamins and minerals (other than iron). Dietary planning guidance for the appropriate replacement of nutrients provided by wheat is warranted.

## Introduction

Celiac disease (CD) is a systemic, autoimmune disease triggered by the ingestion of gluten in genetically susceptible individuals (Leonard et al., 2017). The inflammatory process targets the intestine, leading to progressive villi damage and, consequently, malabsorption of nutrients, particularly iron, folate, calcium and fat-soluble vitamins (Theethira, Dennis & Leffler, 2014). Multiple micronutrient deficiencies are common at diagnosis and frequently persist despite adherence to gluten-free diet (GFD) (Rondanelli et al., 2019). A recent review reported rates of up to 40% for iron and zinc deficiency, 30% vitamin B_12_ deficiency, and 20% folate deficiency in adults with CD with over 2 years of good GFD compliance (Rondanelli et al., 2019). While recovery of nutritional status has been linked to intestinal healing, mucosal healing may be incomplete despite GFD adherence (Theethira, Dennis & Leffler, 2014). For example it has been estimated that only 57–76% of newly diagnosed CD cases achieve intestinal healing following initiation of a GFD (Ludvigsson et al., 2014). Thus, long-term nutritional status may be influenced by refractory disease, inflammatory processes and inadvertent gluten exposure, in addition to dietary choices, and requires monitoring.

A strict, lifelong GFD is the only treatment currently available for CD. This involves the elimination of all gluten-containing foods and ingredients derived from wheat, barley or rye (Vici et al., 2016). This restriction can be problematic as wheat is a major contributor of carbohydrate, dietary fibre, protein, B vitamins and phytochemicals in Western diets (Shewry & Hey, 2015). Wheat also serves as the mandatory fortification vehicle in Canada for iron, folic acid, thiamine, riboflavin and niacin in all refined white (wheat) flour (Government of Canada, 2019a). Conversely, whole wheat and whole grain flours are not enriched, creating a different nutrient profile that provides fibre, magnesium, protein, zinc and selenium (Government of Canada, 2015). Packaged gluten free (GF) grain products such as breads and cereals may be voluntarily enriched by manufacturers, however this practice is not required (Government of Canada, 2019a), and the nutritional quality of such products has been established as inferior compared to fortified wheat staples (Elliott, 2018; Jamieson, Weir & Gougeon, 2018; Kulai & Rashid, 2014). This is likely related to reliance on corn and rice as base ingredients for GF foods, which are high in starch but low in micronutrients and fibre (Pellegrini & Agostoni, 2015). While pseudocereals, legumes and oilseeds may have useful functional, as well as nutritional, properties, incorporation into GF foods may be limited due to cost or availability (Gobbetti et al., 2018). Therefore, in addition to gluten avoidance, nutrients provided by wheat-based staples must be replaced with nutrient-dense food choices to ensure adequacy on this long-term, restrictive diet.

The modern food environment, with an ever-growing abundance of food choices, as well as changing food labels and health claims, is complex (Slater, 2017). For people with CD, the ability to accurately identify safe foods from a nutrition label is necessary for diet adherence (Downey et al., 2015). In fact, adherence and avoiding cross-contamination is the main focus of GFD management in current clinical practice guidelines for CD (Ludvigsson et al., 2014; Rubio-Tapia et al., 2013, Downey et al., 2015), while guidance on healthy GF eating and nutrition education are lacking. Understanding GF nutrient sources in today’s food landscape is important for designing strategies to meet dietary guidelines. While there is a growing body of evidence reporting low nutrients intake for people with CD (Gonzalez et al., 2018; Martin et al., 2013; Wild et al., 2010), few studies have used a dietary pattern approach to analyse food sources of nutrients. To address this gap, a secondary analysis of food records collected from participants on GFD enrolled in the MOVE-C lifestyle intervention pilot study (Dowd et al., 2019) was conducted to identify food sources of energy and nutrients of concern. Findings will inform patterns of dietary adequacy for people requiring a GFD.

## Materials and Methods

### Population selection

Details on participant recruitment and the intervention protocol were previously reported (Dowd et al., 2019). Briefly, a convenience sample of volunteers was recruited through physician referral, online and local advertising. Inclusion criteria were: a self-reported CD diagnosis (through blood test and/or biopsy) or gluten intolerance, physical inactivity, residence in the Calgary (Alberta, Canada) area, and an age of at least 18 years. Thus, participants were on a GFD prior to entering the study. The primary outcomes were quality of life and gut microbiota composition following 12 weeks of exercise training. Diet records were used for secondary analyses. The full study protocol was approved by the University of Calgary Research Ethics Boards (REB 16-17740-MOD4). Informed, voluntary, written consent was obtained prior to participant enrolment. Approval for secondary analysis of data was granted by the St. Francis Xavier University Research Ethics Board (Romeo #23766).

### Data collection

Demographic data were collected through an online questionnaire. Written, self-reported dietary data from 35 participants was collected using 3-day weighed food records on three separate occasions, resulting in a total of eighty 3-day records, or 240 days of food intake. Dietary data was collected before (*n* = 35) and immediately after the 12-week intervention (*n* = 23), with a follow-up assessment 3 months later (*n* = 22). A Registered Dietitian entered all dietary data into the nutrient analysis software FoodWorks (Version 18.0; The Nutrition Company, Long Valley, NJ, USA), linked to the 2015 Canadian Nutrient File (CNF). Missing nutrient values were imputed using the CNF, a brand-specific nutrition fact panel (NFP) or assumed to be zero based on the ingredient list, when appropriate. There were no missing values in the final nutrient data file. Supplements recorded in the food records were excluded from further analysis.

### Data analysis

Willet’s criteria were applied to evaluate plausible energy reporting, and no food records were excluded (Willett, 2012). Descriptive statistics were carried out on nutrients intake. Mean nutrients intake was compared to the Dietary Reference Intakes to provide context in interpreting the food sources of nutrients and whole grain consumption patterns in this sample population. Mean nutrients intake below the Estimated Average Requirement (EAR), recommended daily allowance (RDA), or Adequate Intake, or above the Tolerable Upper Intake Level, were identified as nutrients of concern. After combining all time points, the final food item list contained 4,189 discrete entries. Each food was classified using the Bureau of Nutritional Sciences (BNS) Food Group Codes and Descriptions developed for the Canadian Community Health Survey. Major categories of foods were adapted from the USDA Dietary Source Nutrient Database, as previously reported (Jamieson, Rosta & Gougeon, in press), to reduce 78 BNS groups and sub-groups into eleven main categories for aggregate analyses (Table S1). Food items were classified as reported, rather than disaggregated, as recipe and ingredient-specific data were not available. Combined food items (e.g. pizza, sandwich) were captured under the BNS ‘Mixed dishes’ sub-categories for grains, meat, poultry and fish (MPF), dairy, vegetables, or fruit. Minor adaptations to the BNS categories were made, as previously described (Jamieson, Rosta & Gougeon, in press), to ensure comparability to a previously described Canadian GF population. For each food grouping and sub-category, cumulative nutrient values were determined and expressed as a percentage of total nutrient amount. Food groupings were ranked in order from highest to lowest according to nutrient contribution percentage. All statistical analyses were conducted in StataSE 14.2 (StataCorp, 2015. College Station, TX, USA).

## Results

### Participant characteristics

Participants with valid dietary data included 35 adults (83% female) with CD, residing in Calgary, Alberta. Mean age was 47 ± 11.5 years, body mass index was 28.2 ± 5.2 at the start of the study and average time since diagnosis was 6.7 ± 6.0 years.

### Nutrients intake

No difference in mean energy intake was observed across the three time points so all dietary data was combined for further analyses (Table 1). Macronutrients intake were, on average, 45.2% from carbohydrate, 37.2% from fat and 17.5% from protein. Mean intakes of calcium and vitamin C did not meet the EAR. Mean intakes of iron were between the EAR and RDA for females <51 years and mean dietary fibre intakes fell below the AI. All other mean nutrients intake exceeded the RDA, while mean sodium intake surpassed the UL.

**Table 1:** Mean nutrients of Canadian adults following a gluten-free diet in comparison to Dietary Reference Intakes.

		Mean	SD^b^	Median	EER^c^/EAR^d^	RDA^e^/AI^f^/UL^g^
Energy (kcal)	Females	1,796	453.3	1,714	1,930^c^	–
	Male	1,786	492.7	1,751	2,576^c^	–
Protein (g)	Female	76.9	17.3	77.4	–	46^e^
	Male	77.3	23.7	71.0	–	56^e^
Total fat (g)		73.8	20.7	71.6	–	–
Carbohydrate (g)		204.9	69.9	186.2	100^d^	130^e^
Total sugar (g)		76.9	31.1	74.5	–	–
Dietary fibre (g)	Female	21.3	9.2	19.3	–	25^f^
	Male	17.9	4.8	18.2	–	30–38^f^
Calcium (mg)		769.9	313.3	765.9	800–1,000^d^	1,000–1,200^e^
Iron (mg)	Female < 51 years	10.8	3.2	10.7	8.1^d^	18^e^
	Female ≥ 51 years and males	12.1	4.2	10.5	5–6^d^	8^e^
Sodium (mg)		2,509.1	817.5	2,569	–	1,300–1,500^f^
					–	2,300^g^
Vitamin C (mg)	Female	116.7	81.6	95.2	60^d^	75^e^
	Male	67.0	25.1	73.0	75^d^	90^e^
Vitamin A (µg RAE)	Female	742.8	314.8	688.4	500^d^	700^e^
	Male	811.2	318.8	789.6	625^d^	900^e^

**Notes:**

a Data represent 240 days of records from 35 participants.

b SD, standard deviation.

c EER, estimated energy requirement.

d EAR, estimated average requirement.

e RDA, recommended daily allowance.

f AI, adequate intake.

g UL, tolerable upper limit.

### Food sources of nutrients

Food group sources of energy, protein, iron, carbohydrate, dietary fibre and total sugar are shown in Tables 2 and 3. Food sources of fat, calcium, sodium, vitamin C, vitamin A, cholesterol and saturated fat are available online as Tables S2–S6.

**Table 2:** Food group sources of energy, protein and iron amongst adults eating a gluten-free diet^a^.

Food group	Sub-category^b^	Energy		Protein		Iron	
		Rank	Total (%)	Rank	Total (%)	Rank	Total (%)
Meat, poultry and fish		1	19.5	1	45.1	2	22.6
	Mixed dishes, mainly meat, poultry, fish		4.2		7.2		4.2
	Poultry		4.0		14.1		3.8
	Mixed dishes, mainly luncheon meats, hamburgers or hotdogs		3.3		3.3		3.9
	Luncheon meats and sausages		2.5		3.5		1.7
	Pork		1.9		5.5		1.5
	Beef		1.8		5.5		3.6
	Fish and shellfish		1.5		5.2		2.8
Grain products		2	19.0	3	10.0	1	22.8
	Yeast breads		4.8		1.7		3.1
	Breakfast and hot cereals		4.5		2.7		11.1
	Rice and rice noodles		2.1		1.0		1.6
	Mixed dishes, mainly grain		2.1		1.8		2.9
	Quick breads		1.6		<1		1.4
	Crackers and crispbreads		1.4		<1		1.0
	Pasta		1.2		<1		<1
Dairy Products		3	12.5	2	17.9	8	5.4
	Cheese		4.9		7.9		<1
	Eggs and omelettes		3.0		4.6		3.1
	Yogurt		1.5		2.5		<1
	Milk		1.2		2.1		<1
	Other ‘milk’ beverages		1.0		<1		<1
Desserts and sweets		4	8.9	7	3.3	7	6.4
	Confectionary		3.3		<1		1.8
	Sweet baked goods		2.8		<1		2.2
	Cereal or granola bars		1.1		1.1		1.5
Beverages		5	8.0	5	5.3	6	6.6
	Alcoholic beverages		3.2		<1		1.9
	Mixed beverages		2.4		2.4		<1
	Protein powders and meal replacements		<1		2.3		2.8
Vegetables		6	8.0	4	6.0	3	14.6
	Vegetables, excluding potatoes		2.5		2.8		8.8
	Mixed dishes, mainly vegetables (not potatoes)		1.9		1.5		2.6
	Potatoes, fried or roasted		1.8		<1		1.1
	Potatoes, cooked		1.8		1.0		2.1
Fruit		7	6.0	10	1.8	9	3.8
	Fruit		5.4		1.6		3.4
Legumes, nuts and seeds		8	5.5	6	5.2	4	7.7
	Nuts, seeds, butters and spreads		4.1		3.5		4.3
	Mixed dishes, mainly legumes		<1		<1		2.0
	Legumes (tofu)		<1		<1		1.2
Savoury snacks		9	5.3	9	2.0	10	2.8
	Salty, high fat snacks		3.8		1.4		1.6
	Plain pretzels and popcorn		1.4		<1		1.2
Other foods		10	4.5	8	3.2	5	7.1
	Miscellaneous soups		1.6		2.2		2.6
	Sugars, syrups and preserves		1.5		<1		<1
	Condiments and sauces		1.0		<1		1.2
Fats and oils		11	2.8	11	0.1	11	0.2
	Salad dressings and mayonnaise		1.6		<1		<1
	Fats, oils and spreads		1.2		<1		<1

**Notes:**

a Data represent 240 diet records from *n* = 35 participants.

b Specific percentages are reported only for food group sub-categories that contribute at least 1% of the total nutrient contribution.

**Table 3:** Food group sources of carbohydrate, fibre and total sugar amongst adults eating a gluten-free diet^a^.

Food group	Sub-category^b^	Carbohydrate		Dietary fibre		Total sugar	
		Rank	Total (%)	Rank	Total (%)	Rank	Total (%)
Grain products		1	30.0	1	23.2	5	9.7
	Breakfast and hot cereals		7.3		7.4		4.4
	Yeast breads		7.3		7.0		2.2
	Mixed dishes, mainly grain		2.5		2.6		<1
	Rice and rice noodles		4.0		1.5		<1
	Quick breads		2.6		1.5		1.3
	Pasta		2.2		<1		<1
	Crackers and crispbreads		1.9		1.4		<1
	Rolls, bagels, tortillas, pita, croutons		<1		1.1		<1
Fruit		2	11.9	3	16.8	1	20.6
	Fruit		10.7		16.2		18.1
	Fruit juices		1.0		<1		2.2
Vegetables		3	11.8	2	22.0	7	6.9
	Vegetables, excluding potatoes		4.4		12.4		4.3
	Potatoes, cooked		3.2		3.1		<1
	Potatoes, fried or roasted		2.1		1.9		<1
	Mixed dishes, mainly vegetables (not potatoes)		2.1		4.6		1.9
Desserts and sweets		4	11.1	5	5.9	2	18.6
	Confectionary		4.2		1.7		8.7
	Sweet baked goods		3.5		1.4		5.2
	Cereal or granola bars		1.2		1.9		1.5
	Frozen dairy foods		<1		<1		1.2
	Commercial cookies and biscuits		<1		<1		1.1
Beverages		5	8.2	9	2.2	3	17.4
	Mixed beverages		3.2		<1		8.2
	Soft drinks, regular		1.4		<1		3.7
	Alcoholic beverages		1.3		<1		1.4
	Fruit drinks		1.0		<1		2.5
	Protein powders and meal replacements		<1		1.3		<1
Other foods		6	7.0	6	5.1	4	11.6
	Sugars, syrups and preserves		3.6		<1		8.0
	Miscellaneous soups		1.6		1.9		1.3
	Condiments and sauces		1.2		1.3		1.9
Meat, poultry, fish		7	6.2	8	4.9	8	2.7
	Mixed dishes, mainly luncheon meats, hamburger, hotdog		3.1		2.1		1.2
	Mixed dishes, mainly meat, poultry or fish		2.8		2.6		1.4
Savoury snacks		8	5.5	7	5.0	10	1.4
	Salty, high fat snacks		3.8		2.4		<1
	Plain pretzels and popcorn		1.7		2.5		<1
Dairy Products		9	4.9	10	1.5	6	9.0
	Yogurt		1.6		<1		3.5
	Milk		1.2		<1		3.3
Legumes, nuts and seeds		10	3.0	4	13.3	9	1.6
	Nuts, seeds, butters and spreads		1.5		9.4		<1
	Mixed nuts and trail mixes		<1		<1		<1
	Mixed dishes, mainly legumes		<1		1.8		<1
	Legumes (tofu)		<1		1.8		<1
Fats and oils		11	0.4	11	0.0	11	0.5

**Notes:**

a Data represent 240 diet records from *n* = 35 participants.

b Specific percentages are reported only for food group sub-categories that contribute at least 1% of the total nutrient contribution.

Top energy sources included MPF (19.5%), GF grain products (19.0%), dairy products (12.5%), desserts and sweets (8.9%) and beverages (8.0%) (Table 2). Although MPF was the highest ranked food group for energy, two of the top sub-categories including mixed dishes, which also incorporated GF breads (e.g. hamburgers), potatoes and/or vegetables, grains (e.g. rice or noodle stir fry). Poultry was the highest ranked non-mixed MFP sub-category for energy. For grain products, yeast breads, breakfast and hot cereals, mixed grain dishes (pasta and rice-based), and rice were the largest energy contributors, in descending order. Legumes, nuts and seeds (LNS) contributed 5.5% of energy.

Top sources of protein were MPF (45.1%), dairy products (17.9%), followed by grain products (10.1%) (Table 2). The greatest food sources of iron were grain products (22.8%) (primarily cereals), MPF (22.6%), vegetables (14.6%) and LNS (7.7%) in descending order (Table 2). Breakfast and hot cereals included oatmeal or oats (35.6 % of items) and seven commercial ready-to-eat cereal brands (three of which were fortified with vitamins and minerals). Yeast breads included 13 different commercial brands and nine generic CNF items. Four of the 13 commercial bread brands were enriched. Mixed grain dishes included 10 fried rice dishes, seven rice and cheese dishes, five pasta-based dishes (with cheese, vegetables or meats). As a percentage of all items, the LNS category included 33.9% of items as seeds (e.g. psyllium, hemp, chia, flax and pumpkin), 27.0% nut items (e.g. almond, cashew, walnut), 23.8% nut butters (mainly from peanut), 6.4% chickpea or hummus and 3.7% beans.

In descending order, the top sources of carbohydrates were grain products (30.0%), fruit (11.9%), vegetables (11.8%) and desserts and sweets (11.1%) (Table 3). Breakfast and hot cereals, yeast breads, mixed grain dishes, and rice were the greatest carbohydrate contributors among grain products. LNS contributed 3.0% of carbohydrate intake. The top sources of dietary fibre were grain products (23.2%) (from breakfast and hot cereals, yeast breads and mixed grain dishes), vegetables (22.0%) and fruit (16.8%) (Table 3). LNS ranked fourth in fibre contribution (13.3%), mainly in the form of nuts and nut butters. The top ranked sources of sugar were fruit (20.6%), desserts and sweets (18.6%) and beverages (17.4%) (Table 3). Grain products ranked fifth as a source of sugar (9.7%).

## Discussion

The GF food environment is complex and rapidly changing, with new and diverse food options for emerging populations of consumers and patients seeking GF foods. While the poor diet quality of packaged GF foods has been previously established (Elliott, 2018; Jamieson, Weir & Gougeon, 2018; Kulai & Rashid, 2014), actual food sources of nutrients in the GFD have not been thoroughly explored. Thus, this study presents novel findings on the top nutrient sources for a sample of Canadian adults diagnosed with CD or gluten intolerance.

In agreement with GFD assessment studies (Vici et al., 2016), lower mean intakes of dietary fibre, calcium and iron (for women) from food sources were observed in this study, but most mean nutrient intakes met recommendations. Macronutrient balance was shifted from the recommended ranges towards lower energy intake from carbohydrate (with half of participants consuming less than 45%) and higher energy intake from fat (with over half of participants consuming more than 35%). This is exemplified by lower mean intake of dietary fibre and the higher ranking of energy from MPF over grains. The mean 45% energy from carbohydrate is lower than previously observed in Nova Scotia (NS) adults consuming a GFD (50%), despite a similar sex balance in study samples (Jamieson, Rosta & Gougeon, in press). Similarly, a study of diet-experienced people with CD (*n* = 55) in Australia reported 45.8 ± 0.7% energy as carbohydrate (Shepherd & Gibson, 2013), whereas a British diet study (*n* = 49) reported a 48% average (Wild et al., 2010). Thus, average carbohydrate intake in the present study is at the lower end of observed intakes in CD. This may partly be related to the regional dietary pattern which trends toward lower relative carbohydrate intakes than the national average (Statistics Canada, 2019). Additionally, this could reflect other participant characteristics such as lack of food skills or literacy with respect to wheat replacement or lack of support and dietary guidance for those requiring GFD, as previously noted (Jamieson & Gougeon, 2019).

Approximately two-thirds (67.9%) of the energy consumed by this sample was derived from five food groups (MPF, grain products, dairy products, desserts and sweets and beverages). Notably, this resulted in a higher ranking of MPF and lower ranking of fruits and vegetables in this sample compared to the NS GF study (Jamieson, Rosta & Gougeon, in press) and was reflected in the nutrients with lower mean intakes (fibre, iron). The high-ranking contribution of dairy foods (third) to energy intake is not reflected in the mean calcium intake observed. However, this may be explained by the emphasis on cheese and eggs (4.9% and 3.0% of energy, respectively) and lesser emphasis on yogurt and milk. LNS ranked quite low (eighth) on the contribution to energy intake, in agreement with the NS GF Study (seventh) (Jamieson, Rosta & Gougeon, in press).

Of all grain products, cereals were the top contributor of carbohydrate, dietary fibre and iron in agreement with the NS GF Study (Jamieson, Rosta & Gougeon, in press). At the same time, grain products were fifth in contribution to total sugar intake, with cereals as the top source. Cereal as a top source of fibre is likely related to frequent consumption of oats and unenriched, ready-to-eat cereal brands which tended to be made from whole grains and ‘natural’ ingredients. In contrast, enriched ready-to-eat cereals were also frequently consumed, likely contributing to micronutrient intake, but not fibre. Thus, GF cereals appear to represent key foods with two distinct nutrient profiles, varying with enrichment practices and fibre-rich ingredients. Notably, vegetables, fruits and nuts were greater dietary fibre contributors than cereals.

Gluten-free breads were also a key contributor to carbohydrate and dietary fibre, and to a lesser extent, iron intakes, despite being primarily unenriched (89.3%). This may be related to the growing food trend to incorporate ancient grains and seeds in the North American market (Roesler, 2018). Thus, while GF breads often use rice or starch as the first ingredient, other whole grain flours (e.g. amaranth, quinoa) and seeds (e.g. chia, flax) may be added as secondary ingredients, improving nutrient density. However, while ranking highly amongst grain products, the absolute contribution of iron was still relatively small for breads.

A mixed grain dish category (rice or pasta-based) was the other top grain sub-category source of carbohydrate, dietary fibre and iron. However, nutrient contributions from this category are difficult to interpret since additional ingredients (e.g. meats, vegetables) are also included. In Canada, the enrichment of rice and non-wheat pasta is voluntary (Government of Canada, 2019a) and thus not a reliable micronutrient source. The selection of brown rice, brown rice noodles, and whole grain-based GF pastas could enhance fibre and select micronutrient (e.g. magnesium, zinc) intakes (Government of Canada, 2015). In addition, non-grain key contributors of iron (e.g. LNS and select vegetables) could be emphasised to improve intakes for those following the GFD. In fact, these suggestions align well with Canada’s Food Guide, which emphasises a central role for vegetables and fruits, along with whole grains and plant-based protein foods such as beans, lentils, nuts and seeds (Government of Canada, 2019b). In the present sample, LNS made a small contribution (<8%) to energy, carbohydrate, iron and protein, but a moderate contribution (13.3%) to dietary fibre. Most LNS choices in the present study were from various seeds, nuts and seed/nut butters, contributing more fat than fibre. In the present study, and in the NS GF Study (Jamieson, Rosta & Gougeon, in press), infrequent legume consumption was observed, an especially rich plant source of naturally occurring vitamins and iron (Government of Canada, 2015).

This study has several important limitations. Due to the nature of convenience samples, the dietary patterns observed cannot be generalised. However, the large number of diet records collected over several time points helps to counter this problem. The lack of ingredient-specific data was a limiting factor in food categorisation. The lack of brand specific GF nutrient composition data (e.g. B vitamins, minerals) limited the number of nutrients that could be reliably analysed. A control group was not available for comparison purposes, however some national data on food sources of nutrients are available for the general Canadian population (Garrigeut, 2011; Langlois & Garrigeut, 2011; Statistics Canada, 2017). As with any food coding system, results are affected by categorisation choices and thus, comparisons between studies should be done with caution. However, this study did follow the same protocol as the NS GF Study and is comparable in that respect. Finally, there were some missing diet records (19%) which may have influenced the types of foods reported in this sample.

## Conclusions

Although carbohydrate accounted for only 45% of energy intake, on average, in this sample of adults following a GFD, grain products were still a top source of nutrients including carbohydrate, dietary fibre and iron. Top grain contributors included cereals and yeast breads, despite few of the brands reported being fortified or enriched with micronutrients. Thus, while GF grains are a source of fibre, they are less reliable as key sources of vitamins and minerals. Given this and the lack of nutrient composition data for micronutrients (other than iron) in GF foods, it would be prudent to re-evaluate enrichment and fortification policies, which presently are optional for GF flours in Canada. In addition, improvements in the nutritional quality of GF food formulations could contribute to nutrient adequacy for those requiring long-term GFD. Findings also suggest that legumes, a good source of folate, potassium, minerals, fibre and protein, were not a key nutrient source in this study. As people with CD are at increased risk of vitamin and mineral deficiency (Rondanelli et al., 2019), nutritional adequacy of the GFD will require dietary planning and appropriate replacement of nutrients provided by enriched wheat products. Without reliable exposures to key micronutrients through population-level fortification, nutritional monitoring and dietary guidance from a registered dietitian appears even more critical. Further research is needed on nutrient composition data in novel GF foods and ingredients, as well as in regard to the accessibility of registered dietitians to support people requiring long-term GFD management.

## Supplemental Information

10.7717/peerj.9590/supp-1 Supplemental Information 1 Tables S1–S6.

10.7717/peerj.9590/supp-2 Supplemental Information 2 Raw data: Gluten-free food sources of nutrients. Nutrient contents for each food item is presented, with corresponding food group codes for analyses.

## Additional Information and Declarations

### Data Availability

The following information was supplied regarding data availability: The raw nutrient data for each food item reported is available in File S1.
