# Supplementary material for: Food sources of energy and nutrients among Canadian adults following a gluten-free diet
Source: PeerJ. 2020 Jul 27;8:e9590. doi: 10.7717/peerj.9590 (PMC7391969; doi:10.7717/peerj.9590)
Supplement: Supplemental Information 1 [file peerj-08-9590-s001.docx]

**SUPPLEMENTAL TABLES S1-S6**

Food sources of energy and nutrients among Canadian adults following a gluten-free diet

Table S1 Bureau of Nutritional Sciences (BNS) Food Groupings adapted for the Gluten-free Diet

| **Grain products** | **Vegetables** | **Savoury snacks** |
| --- | --- | --- |
| Pasta | Vegetables, excluding potatoes | Plain pretzels and popcorn  Salty, high fat snacks^m^ |
| Rice and rice noodles | Potatoes, cooked |  |
| Cereal grains and flours | Potatoes, fried or roasted | **Desserts & sweets** |
| Yeast breads | Mixed dishes, mainly potatoes^h^ | Commercial cookies and biscuits |
| Rolls, bagels, tortillas, pita, croutons | Mixed dishes, mainly vegetables | Cereal or granola bars |
| Crackers and crispbreads |  | Commercial baked goods^n^ |
| Quick breads | **Fruit** | Frozen dairy foods |
| Breakfast and hot cereals | Fruit | Confectionary^o^ |
| Mixed dishes, mainly grain | Fruit juices^k^ | Sweet baked goods^p^ |
|  | Mixed dishes, mainly fruit^j^ |  |
| **Dairy products & eggs** | **Meat, poultry, fish** | **Beverages** |
| Milk | Beef | Soft drinks, regular |
| Other ‘milk’ drinks^d^ | Pork | Soft drinks, artificially sweetened |
| Cream | Poultry | Fruit drinks |
| Cheese | Luncheon meats and sausages | Tea and coffee |
| Yogurt | Fish and shellfish | Water |
| Eggs and omelettes | Mixed dishes, mainly meat, poultry or fish | Protein powders |
| Mixed dishes, mainly dairy^e^ | Mixed dishes, mainly luncheon meats, hamburgers or hotdogs | Mixed beverages^q^ |
| **Fats & oils** | **Legumes, nuts, seeds** | **Other foods** |
| Fats, oils and spreads^f^ | Nuts | Condiments and sauces^r^ |
| Salad dressings and mayonnaise | Seeds | Sugars, syrups and preserves |
|  | Nut spreads and butters | Seasonings and spices |
|  | Legumes and legume-based foods (tofu) | Miscellaneous soups |
|  | Nut and seed mixed foods (trail mix) |  |
| ^a^includes rice crackers and rice cakes  ^b^includes muffins, English muffins, pancakes, waffles and commercial mixes of these products  ^c^includes commercial and homemade mixed dishes primarily containing rice, pasta, grains, cereals, flours or breads; includes breakfast sandwiches containing eggs and meats  ^d^includes soy, almond, coconut and rice-based beverages  ^e^includes cream-based dips and processed cheese spreads  ^f^includes butter, margarine, vegetable oils and animal fats  ^g^includes beans, cruciferous vegetables, carrots, sweet potatoes, celery, corn, lettuces, leafy greens, mushrooms, onions and garlic, peas, squashes, tomatoes, vegetable juices, asparagus, cucumbers, beets and turnips)  ^h^includes dishes made with boiled, mashed or baked potatoes, French fries and hash brown potatoes  ^i^includes salads, other baked or mixed vegetables (excluding potatoes)  ^j^includes raw, cooked, frozen and canned fruits  ^k^includes orange juices fortified with calcium and vitamin D  ^j^includes fruit-based smoothies, applesauce and cooked, sweetened rhubarb and cranberries  ^m^includes potato chips, corn chips, salted and oil-popped popcorn  ^n^includes commercially prepared cakes, pies, danishes, doughnuts and other pastries  ^o^includes candies, gums, popsicles, jello, pudding mixes, chocolate bars, chocolate covered candies  ^p^includes homemade or bakery-made cakes, cookies, pastries, muffins, pies, squares, bars, crepes, sweet breads  ^qi^ncludes chocolate milk, sweetened teas, and milk-based coffee drinks  ^r^includes tomato-based sauces, pesto, mustards, chutney, and cranberry sauce | | |

Table S2 Food Group Sources of Fat Among Adults following a Gluten-free diet^a^

| **Main Food Group,** Sub-Category**^b^** | % Total Fat (cumulative) |  |
| --- | --- | --- |
| **MEAT, POULTRY, FISH** | **23.8 (23.8)** |  |
| Luncheon meats and sausages | 4.9 (4.9) |  |
| Mixed dishes, mainly meat, poultry or fish | 4.6 (9.5) |  |
| Poultry | 3.8 (13.3) |  |
| Mixed dishes, mainly luncheon meats, hamburgers or hotdogs | 3.6 (16.9) |  |
| Pork | 2.4 (19.3) |  |
| Beef | 2.2 (21.5) |  |
| Fish and shellfish | 1.3 (22.8) |  |
| Liver and Liver pates | 1 (23.8) |  |
| **DAIRY PRODUCTS** | **19.2 (43.1)** |  |
| Cheese | 9.1 (9.1) |  |
| Eggs and omelettes | 5.3 (14.4) |  |
| Other ‘milk’ beverages | 1.5 (15.9) |  |
| Cream | 1.4 (17.4) |  |
| **GRAIN PRODUCTS** | **11.1 (54.2)** |  |
| Yeast breads | 4.3 (4.3) |  |
| Breakfast and hot cereals | 2.1 (6.4) |  |
| Mixed dishes, mainly grain | 1.7 (8) |  |
| Crackers and crispbreads | 1.2 (9.2) |  |
| **LEGUMES, NUTS and SEEDS** | **9.8 (63.9)** |  |
| Nuts, seeds, nut/seed butters/spreads | 8.4 (8.4) |  |
| **DESSERTS and SWEETS** | **8.7 (72.7)** |  |
| Confectionary | 3 (3) |  |
| Sweet baked goods | 2.9 (5.9) |  |
| Cereal or granola bars | 1.2 (7.1) |  |
| **FATS & OILS** | **6.9 (79.5)** |  |
| Fats, oils and spreads | 4.4 (4.4) |  |
| Salad dressings and mayonnaise | 2.5 (6.9) |  |
| **SAVOURY SNACKS** | **6.8 (86.3)** |  |
| Salty, high fat snacks | 5.2 (5.2) |  |
| Plain pretzels and popcorn | 1.6 (6.8) |  |
| **VEGETABLES** | **5.6 (91.9)** |  |
| Potatoes, fried or roasted | 2.2 (2.2) |  |
| Mixed dishes, mainly vegetables (not potatoes) | 2 (4.2) |  |
| **OTHER FOODS** | **3 (94.9)** |  |
| Miscellaneous soups | 1.4 (1.4) |  |
| Condiments and sauces | 1.3 (2.7) |  |
| **FRUIT** | **2.7 (97.6)** |  |
| Fruit | 2.5 (2.5) |  |
| **BEVERAGES** | **2.4 (100)** |  |
| Mixed beverages | 1.6 (1.6) |  |
| ^a^Data represent 240 diet records from n=33 participants.  ^b^Specific percentages are reported only for food group sub-categories that contribute at least 1% of the total nutrient contribution. | |  |
|  |  |  |
|  |  |  |
|  |  |  |
|  |  |  |
|  |  |  |
|  |  |  |

Table S3 Food Group Sources of Calcium Among Adults following a Gluten-free diet ^a^

| **Main Food Group,** Sub-Category**^b^** | % Total Calcium (cumulative) |
| --- | --- |
| **DAIRY PRODUCTS** | **41.5 (41.5)** |
| Cheese | 20.9 (20.9) |
| Milk | 7.7 (28.6) |
| Yogurt | 4.6 (33.2) |
| Other ‘milk’ beverages | 4.5 (37.7) |
| Eggs and omelettes | 2 (39.8) |
| Cream | 1.5 (41.2) |
| **BEVERAGES** | **13.1 (54.6)** |
| Mixed beverages | 7.2 (7.2) |
| Protein powders | 2.1 (9.3) |
| Water | 1.8 (11.1) |
| **GRAIN PRODUCTS** | **11 (65.6)** |
| Breakfast and hot cereals | 3.7 (3.7) |
| Mixed dishes, mainly grain | 2.6 (6.4) |
| Quick breads | 1.6 (8) |
| Yeast breads | 1.2 (9.2) |
| **VEGETABLES** | **8.7 (74.3)** |
| Vegetables, excluding potatoes | 5.4 (5.4) |
| Mixed dishes, mainly vegetables (not potatoes) | 1.9 (7.3) |
| **MEAT, POULTRY, FISH** | **7.5 (81.8)** |
| Mixed dishes, mainly luncheon meats, hamburgers or hotdogs | 3.3 (3.3) |
| Mixed dishes, mainly meat, poultry or fish | 1.4 (4.7) |
| **DESSERTS and SWEETS** | **4.6 (86.4)** |
| Confectionary | 1.6 (1.6) |
| Sweet baked goods | 1.2 (2.8) |
| **OTHER FOODS** | **3.7 (90.1)** |
| Seasonings and spices | 1.7 (1.7) |
| **FRUIT** | **3.7 (93.8)** |
| Fruit | 2.6 (2.6) |
| Fruit juices | 1 (3.6) |
| **LEGUMES, NUTS and SEEDS** | **3.6 (97.4)** |
| Nuts, seeds, nut/seed butters/spreads | 2.5 (2.5) |
| **SAVOURY SNACKS** | **2.3 (99.7)** |
| Salty, high fat snacks | 2.1 (2.1) |
| **FATS & OILS** | **0.3 (100)** |

^a^Data represent 240 diet records from n=33 participants.

^b^Specific percentages are reported only for food group sub-categories that contribute at least 1% of the total nutrient contribution.

Table S4 Food Group Sources of Sodium Among Adults following a Gluten-free diet ^a^

| **Main Food Group,** Sub-Category | % Total Sodium (cumulative) |
| --- | --- |
| **MEAT, POULTRY, FISH** | **25.4 (25.4)** |
| Mixed dishes, mainly meat, poultry or fish | 7.2 (7.2) |
| Luncheon meats and sausages | 5.4 (12.6) |
| Mixed dishes, mainly luncheon meats, hamburgers or hotdogs | 4.9 (17.5) |
| Pork | 4.1 (21.6) |
| Fish and shellfish | 1.7 (23.3) |
| Poultry | 1.4 (24.7) |
| **GRAIN PRODUCTS** | **17.1 (42.6)** |
| Yeast breads | 6 (6) |
| Mixed dishes, mainly grain | 3.2 (9.2) |
| Breakfast and hot cereals | 2.8 (12) |
| Quick breads | 2.7 (14.6) |
| **DAIRY PRODUCTS** | **15.3 (57.8)** |
| Cheese | 8.1 (8.1) |
| Eggs and omelettes | 4.4 (12.5) |
| **OTHER FOODS** | **12.8 (70.6)** |
| Condiments and sauces | 5.5 (5.5) |
| Miscellaneous soups | 5.2 (10.8) |
| Seasonings and spices | 1.6 (12.4) |
| **VEGETABLES** | **11.2 (81.8)** |
| Mixed dishes, mainly vegetables (not potatoes) | 5.5 (5.5) |
| Vegetables, excluding potatoes | 2.8 (8.3) |
| Potatoes, fried or roasted | 1.8 (10.1) |
| Potatoes, cooked | 1.1 (11.2) |
| **SAVOURY SNACKS** | **4.8 (86.6)** |
| Salty, high fat snacks | 3.7 (3.7) |
| Plain pretzels and popcorn | 1.1 (4.8) |
| **DESSERTS and SWEETS** | **3.8 (90.4)** |
| Sweet baked goods | 1.8 (1.8) |
| **LEGUMES, NUTS and SEEDS** | **3.3 (93.7)** |
| Mixed dishes, mainly legumes | 2 (2) |
| **BEVERAGES** | **3.3 (97)** |
| Mixed beverages | 1.1 (1.1) |
| **FATS & OILS** | **2.1 (99.1)** |
| Salad dressings and mayonnaise | 1.6 (1.6) |
| **FRUIT** | **0.9 (100)** |
| ^a^Data represent 240 diet records from n=33 participants.  ^b^Specific percentages are reported only for food group sub-categories that contribute at least 1% of the total nutrient contribution. | |

Table S5 Food Group Sources of Vitamin C Among Adults following a Gluten-free diet ^a^

| **Main Food Group,** Sub-Category | % Total Vitamin C (cumulative) |
| --- | --- |
| **VEGETABLES** | **43.2 (43.2)** |
| Vegetables, excluding potatoes | 33.6 (33.6) |
| Mixed dishes, mainly vegetables (not potatoes) | 4.7 (38.3) |
| Potatoes, cooked | 3.7 (41.9) |
| Potatoes, fried or roasted | 1.3 (43.2) |
| **FRUIT** | **31.1 (74.4)** |
| Fruit | 25.3 (25.3) |
| Fruit juices | 5.6 (30.9) |
| **BEVERAGES** | **7.4 (81.8)** |
| Fruit drinks | 6 (6) |
| **MEAT, POULTRY, FISH** | **6.1 (87.9)** |
| Mixed dishes, mainly meat, poultry or fish | 3.9 (3.9) |
| Mixed dishes, mainly luncheon meats, hamburgers or hotdogs | 1.5 (5.4) |
| **OTHER FOODS** | **3.8 (91.7)** |
| Seasonings and spices | 1.4 (1.4) |
| Condiments and sauces | 1.2 (2.6) |
| **DAIRY PRODUCTS** | **3.7 (95.4)** |
| Other ‘milk’ beverages | 3 (3) |
| **DESSERTS and SWEETS** | **2.4 (97.8)** |
| Confectionary | 1.7 (1.7) |
| **GRAIN PRODUCTS** | **1.1 (98.9)** |
| **SAVOURY SNACKS** | **0.6 (99.5)** |
| **LEGUMES, NUTS and SEEDS** | **0.5 (99.9)** |
| **FATS & OILS** | **0.1 (100)** |

^a^Data represent 240 diet records from n=33 participants.

^b^Specific percentages are reported only for food group sub-categories that contribute at least 1% of the total nutrient contribution.

Table S6 Food Group Sources of Vitamin A (RAE) Among Adults following a Gluten-free diet ^a^

| **Main Food Group,** Sub-Category | % Total Vitamin A (cumulative) |
| --- | --- |
| **VEGETABLES** | **41.7 (41.7)** |
| Vegetables, excluding potatoes | 34.3 (34.3) |
| Mixed dishes, mainly vegetables (not potatoes) | 7 (41.3) |
| **DAIRY PRODUCTS** | **22.1 (63.8)** |
| Cheese | 7.2 (7.2) |
| Eggs and omelettes | 7 (14.2) |
| Milk | 4 (18.2) |
| Other ‘milk’ beverages | 1.6 (19.8) |
| Cream | 1.4 (21.2) |
| **MEAT, POULTRY, FISH** | **9.4 (73.2)** |
| Mixed dishes, mainly meat, poultry or fish | 4 (4) |
| Fish and shellfish | 1.9 (5.9) |
| Mixed dishes, mainly luncheon meats, hamburgers or hotdogs | 1.4 (7.3) |
| Liver and Liver pates | 1.1 (8.4) |
| **BEVERAGES** | **8.3 (81.5)** |
| Protein powders | 5.3 (5.3) |
| Mixed beverages | 2.8 (8.2) |
| **GRAIN PRODUCTS** | **4.6 (86)** |
| Breakfast and hot cereals | 2.8 (2.8) |
| Mixed dishes, mainly grain | 1.5 (4.3) |
| **OTHER FOODS** | **4.4 (90.4)** |
| Miscellaneous soups | 3.1 (3.1) |
| **FATS & OILS** | **3.1 (93.5)** |
| Fats, oils and spreads | 2.9 (2.9) |
| **DESSERTS and SWEETS** | **2.9 (96.5)** |
| Sweet baked goods | 1.2 (1.2) |
| **FRUIT** | **2.6 (99)** |
| Fruit | 2.1 (2.1) |
| **SAVOURY SNACKS** | **0.9 (99.9)** |
| **LEGUMES, NUTS and SEEDS** | **0.1 (100)** |

^a^Data represent 240 diet records from n=33 participants.

^b^Specific percentages are reported only for food group sub-categories that contribute at least 1% of the total nutrient contribution.
